# Limited positional differences in lower-limb muscle contractile properties among female football players

**DOI:** 10.3389/fspor.2026.1915995

**Published:** 2026-08-07

**Authors:** Jaroslav Sučka, Frederika Pajonková, Pavol Čech, Rút Lenková

**Affiliations:** 1 Department of Educology of Sports, Faculty of Sports, University of Presov, Prešov, Slovakia; 2 Department of Sports Educology and Humanistics, Faculty of Sports, University of Presov, Prešov, Slovakia; 3 Department of Sports Kinanthropology, Faculty of Sports, University of Presov, Prešov, Slovakia

**Keywords:** athlete monitoring, neuromuscular assessment, soccer, tensiomyography, women's sport

## Abstract

**Background:**

Contractile properties of the lower-limb muscles are closely associated with high-intensity activities performed during a football match. Several studies have indicated that playing position significantly influences players' physical and performance demands, as well as the intensity of high-speed locomotor activities during match play. Nevertheless, it remains unclear to what extent muscle contractile properties differ according to specific playing positions in women's football. Therefore, the aim of this study was to describe the contractile properties of female football players according to playing position.

**Methods:**

Female football players (*n* = 52) competing in the highest national league were divided into six groups according to playing position: GK—goalkeepers (*n* = 4), FB—fullbacks (*n* = 9), CB—centre-backs (*n* = 8), W—wingers (*n* = 10), M—midfielders (*n* = 13), and F—forwards (*n* = 8). Muscle contractile properties of the *m. biceps femoris*, *m. gastrocnemius medialis*, *m. gluteus maximus*, *m. vastus lateralis*, and *m. vastus medialis* on both the dominant and non-dominant lower limbs were assessed using the parameters contraction time (Tc), maximal radial displacement (Dm), and contraction velocity (Vc), obtained by the non-invasive method of tensiomyography using the TMG S2 system (TMG-BMC Ltd., Slovenia). The Shapiro–Wilk test indicated that the data were not normally distributed. Differences in contractile properties according to playing position were evaluated using the Kruskal–Wallis analysis of variance (K–W ANOVA), followed by Dunn's *post hoc* test with Bonferroni correction. Holm–Bonferroni correction was subsequently applied to account for multiple comparisons across all analyzed outcomes.

**Results:**

Before adjustment for multiple comparisons, the lowest unadjusted *p*-value was observed for contraction velocity of the dominant vastus lateralis muscle (H = 12.219, *p* = 0.032). However, this finding did not remain statistically significant after Holm–Bonferroni correction. No statistically significant positional differences were observed for the remaining muscles or TMG-derived parameters.

**Conclusions:**

The findings indicate only limited positional differences in lower-limb muscle contractile properties among elite female football players. However, these findings should be interpreted with caution given the relatively small sample size, particularly the goalkeeper subgroup, and the absence of statistically significant differences after adjustment for multiple comparisons. Nevertheless, tensiomyography appears to be a promising non-invasive tool for individualized neuromuscular monitoring when interpreted alongside other performance and clinical assessments.

## 1 Introduction

In recent years, the monitoring of training and match load has become an integral component of elite football. Current research emphasizes the importance of individualized monitoring of both external and internal load in order to optimize performance, enhance recovery, and reduce injury risk (1). Football is a multifaceted team sport that requires well-developed speed, agility, balance, and strength for successful performance at the elite level (2). The game is characterized by dynamic movements, including short and long sprints, explosive reactions, and rapid changes of direction. During directional changes, athletes must generate eccentric force to decelerate and concentric force to accelerate in a new direction (3). These frequent high-intensity actions place substantial demands on the hamstring muscles, which are often required to function under conditions of extreme stretch. Consequently, hamstring injuries are among the most common muscle injuries in football and typically occur during high-speed running, acceleration, and deceleration actions (4). Sprinting and acceleration are decisive actions in modern football while simultaneously contributing substantially to the accumulation of neuromuscular fatigue throughout a match (5). As a result, the majority of injuries affect the lower extremities, particularly the hamstrings, adductors, quadriceps, and calf muscles. Notably, more than 90% of muscle injuries occur in non-contact situations (6).

The high-intensity demands of football are closely associated with the fundamental contractile properties of lower-limb muscles (7). However, limited information is available regarding the mechanical responses of trained muscles across different training periods and their implications for speed and strength performance. In this context, tensiomyography (TMG) has emerged as a simple and non-invasive method for assessing the mechanical properties of skeletal muscles (8). TMG is a non-invasive neuromuscular assessment technique developed at the University of Ljubljana, Slovenia (9). The method involves the application of an electrical stimulus to the muscle, and the resulting radial displacement caused by muscle contraction is recorded using a digital displacement sensor positioned perpendicular to the muscle belly. TMG has been used to evaluate muscle contractile characteristics (10, 11), muscle tone during atrophy (12), and muscle fiber composition (8, 10, 13). The most assessed TMG parameters include maximal radial displacement (Dm), contraction time (Tc), and contraction velocity (Vc). These variables provide valuable information regarding muscle stiffness, explosiveness, neuromuscular readiness, and overall muscle functional status (8). In recent years, TMG has also been increasingly utilized to monitor contractile properties, as it may help identify fatigue- or overload-induced changes that could increase the risk of muscle injury (14). Accordingly, TMG-derived parameters may represent important indicators of neuromuscular injury risk factors (15).

Several authors have emphasized that findings obtained from male football players cannot be directly extrapolated to female football players (14). Women's football exhibits unique biomechanical and neuromuscular characteristics that may influence movement strategies, muscular activation, and injury occurrence (16). Although the scientific literature on football has expanded considerably in recent years, female players remain underrepresented in neuromuscular diagnostic research, with most assessment protocols still being developed and validated predominantly in male populations (14, 17).

Despite the increasing application of tensiomyography in sports diagnostics, the relationship between muscle contractile properties and playing position among female football players remains insufficiently explored (18). Only a limited number of studies have investigated muscle contractility assessed by TMG in relation to positional differences. Previous research focused on male Spanish football players (19, 20), another study examined contractile characteristics in elite players competing in the Serbian Super League (21). In women's football, research investigating muscle contractile properties using TMG remains scarce. To date, only a few studies have examined the neuromuscular characteristics of female football players, and even fewer have analyzed muscle contractile properties according to playing position (22–25).

Given the limited evidence currently available, a more detailed examination of muscle contractile properties across playing positions in female football players using TMG is needed. Therefore, the aim of the present study was to examine the contractile properties of female football players according to playing position. It was hypothesized that players occupying different positions would exhibit significant differences in TMG-derived parameters due to differences in movement patterns, physiological demands, and performance requirements associated with each playing position.

## 2 Materials and methods

### 2.1 Study design

This study employed a cross-sectional observational design to investigate differences in muscle contractile properties among female football players across playing positions. Assessments were performed during the pre-season period of the 2025/2026 competitive season under standardized laboratory conditions. Muscle contractile properties of selected lower-limb muscles were evaluated using tensiomyography (TMG). The primary outcome variables included contraction time (Tc), maximal radial displacement (Dm), and contraction velocity (Vc). Players were classified into six positional groups (goalkeepers, fullbacks, centre-backs, wingers, midfielders, and forwards), and TMG-derived parameters were compared between playing positions.

### 2.2 Participants

The study sample consisted of 52 female football players competing in the highest national football league in Slovakia. A formal *a priori* power analysis was not performed because the study included all eligible elite female football players available during the data collection period. Data collection was conducted during the pre-season period prior to the start of the 2025/2026 competitive season. The participants had a mean age of 17.19 ± 2.58 years, a mean body height of 165.56 ± 6.31 cm, and a mean body mass of 58.20 ± 8.26 kg. Lower-limb dominance was defined as the preferred kicking leg, as reported by the participants. Of the 52 participants, 45 (86.5%) reported the right lower limb as dominant, whereas 7 (13.5%) reported the left lower limb as dominant. Based on their playing position, the players were categorized into six groups: goalkeepers (GK; *n* = 4), fullbacks (FB; *n* = 9), centre-backs (CB; *n* = 8), wingers (W; *n* = 10), midfielders (M; *n* = 13), and forwards (F; *n* = 8). Eligibility for participation required a minimum of five years of competitive football experience. All participants were free from musculoskeletal injury and reported good health status at the time of testing. To minimize the potential influence of acute fatigue on neuromuscular performance, players were instructed to avoid strenuous physical activity for at least 48 h prior to testing.

Before participation, all players received a detailed explanation of the study objectives and experimental procedures and subsequently provided written informed consent. All research procedures were conducted in accordance with the ethical principles outlined in the Declaration of Helsinki. Ethical approval for the study was granted by the Ethics Committee for Research of the University of Prešov, Slovakia (approval number: ECUP062024PO).

### 2.3 Procedure

Prior to the assessment of muscle contractile properties, anthropometric measurements and body composition analysis were performed. Body height was measured using a digital stadiometer (BSM 170, Biospace Co., Ltd., Seoul, South Korea). Body mass and body composition variables were assessed using multifrequency bioelectrical impedance analysis using the InBody 720 device (Biospace Co., Ltd., Seoul, South Korea).

Muscle contractile properties of the biceps femoris (BF), gastrocnemius medialis (GcM), gluteus maximus (GM), vastus lateralis (VL), and vastus medialis (VM) were evaluated bilaterally on both the dominant and non-dominant lower limbs using tensiomyography (TMG), a non-invasive method for neuromuscular assessment. Measurements were performed using the TMG-S2 system (TMG-BMC Ltd., Ljubljana, Slovenia). The following TMG-derived parameters were analyzed: contraction time (Tc), maximal radial displacement (Dm), and contraction velocity (Vc).

Measurements were performed according to a standardized TMG protocol under identical laboratory conditions. Muscle assessments were conducted in a standardized sequence, with the dominant lower limb evaluated first, followed by the non-dominant lower limb. Within each limb, muscles were assessed in the following order: vastus lateralis, vastus medialis, gluteus maximus, biceps femoris, and gastrocnemius medialis. All measurements were performed by the same certified physiotherapist to ensure consistency throughout the testing procedure. Muscle contractions were elicited using a single electrical stimulus with a pulse duration of 1 ms. The initial stimulation intensity was set at 10 mA and progressively increased in 20 mA increments until a maximum intensity of 100 mA was reached. A 10-second rest interval was maintained between consecutive stimuli to minimize the influence of muscle fatigue. The reliability of the TMG method has been demonstrated in previous validation studies, reporting excellent reproducibility (ICC = 0.92–0.97; CV = 2.7%–4.7%) (26). Furthermore, the reliability of TMG measurements for lower-limb muscles has been confirmed in previous validation studies, demonstrating good-to-excellent intra-rater and inter-rater reliability (27–30).

Contraction velocity (Vc), considered an indicator of the functional contractile capacity of skeletal muscle, was calculated according to a previously published equation (26):

Vc=Dm/(Td+Tc)

Muscle contractions were elicited involuntarily through the application of a single electrical stimulus with a pulse duration of 1 ms. Muscle identification and electrode placement were performed by a certified physiotherapist in accordance with the standardized procedures recommended by the device manufacturer. Tensiomyographic measurements were carried out in accordance with a previously published protocol (22).

### 2.4 Statistical analyses

The normality of data distribution was assessed using the Shapiro–Wilk test. As the assumption of normality was violated, data were presented as median (Me) and quartile deviation (QD). Differences in muscle contractile properties between playing positions were analyzed using the Kruskal–Wallis H test (K–W ANOVA). Following a significant Kruskal–Wallis test, pairwise comparisons were performed using Dunn's *post hoc* test with Bonferroni correction. To control the family-wise error rate associated with multiple statistical testing across all analyzed outcomes, Holm–Bonferroni correction was subsequently applied to the obtained *p*-values. Effect size was quantified using eta squared (*η*^2^).

The magnitude of the effect size was interpreted using previously published thresholds (27) as follows: *η*^2^ = 0.01 (small effect), *η*^2^ = 0.06 (moderate effect), and *η*^2^ = 0.14 (large effect). When significant differences were identified, *post hoc* pairwise comparisons were performed using the Bonferroni correction. Statistical significance was set at *p* < 0.05.

All statistical analyses were performed using IBM SPSS Statistics software (Version 27.0; IBM Corp., Armonk, NY, USA).

## 3 Results

The contractile properties of the biceps femoris, gastrocnemius medialis, and gluteus maximus muscles are presented in Table 1. Descriptive differences were observed across playing positions. However, none of these differences reached statistical significance. Inter-limb symmetry indices are presented in Table 1. The Kruskal–Wallis analysis did not reveal statistically significant differences between playing positions for contraction time (Tc), maximal radial displacement (Dm), contraction velocity (Vc), or symmetry indices in the biceps femoris, gastrocnemius medialis, and gluteus maximus muscles (all *p* > 0.05).

**Table 1:** Contractile properties of the biceps femoris, gastrocnemius medialis, and gluteus maximus muscles according to playing position.

parameter			GK	FB	CB	W	M	F	K-W Anova		
			Me (QD)						h	p	*η*2
BF	D	tc	48.9 (4.9)	35.1 (2.5)	43.0 (14.7)	35.0 (12.9)	42.9 (6.3)	44.7 (16.1)	2.599	0.762	0.001
		dm	6.5 (1.9)	3.1 (3.3)	4.4 (3.2)	5.6 (1.3)	6.8 (1.3)	6.0 (2.0)	5.159	0.397	0.003
		vc	0.08 (0.03)	0.05 (0.04)	0.07 (0.03)	0.07 (0.01)	0.11 (0.01)	0.07 (0.02)	8.704	0.121	0.080
	N	tc	46.8 (7.9)	38.7 (3.9)	41.1 (15.2)	35.1 (4.8)	43.3 (4.2)	39.1 (12.4)	4.109	0.534	0.001
		dm	5.1 (3.1)	6.7 (2.9)	7.2 (3.0)	4.4 (2.1)	7.8 (1.9)	5.9 (1.1)	6.195	0.288	0.026
		vc	0.07 (0.04)	0.08 (0.03)	0.08 (0.02)	0.06 (0.03)	0.10 (0.02)	0.08 (0.01)	5.600	0.347	0.013
		sym	79.4 (5.8)	78.5 (12.7)	77.2 (11.0)	88.9 (4.1)	83.6 (6.2)	77.7 (11.3)	4.865	0.433	0.001
GcM	D	tc	32.3 (11.3)	27.1 (0.3)	26.9 (4.1)	24.9 (1.3)	26.1 (2.5)	30.1 (5.0)	6.644	0.249	0.036
		dm	3.9 (1.6)	2.7 (0.5)	3.2 (0.7)	3.5 (0.5)	3.4 (1.0)	3.5 (0.6)	2.448	0.784	0.001
		vc	0.07 (0.02)	0.05 (0.01)	0.06 (0.01)	0.07 (0.01)	0.07 (0.02)	0.06 (0.01)	4.741	0.448	0.001
	N	tc	25.4 (4.2)	31.0 (9.5)	25.9 (4.5)	23.7 (3.1)	24.7 (1.6)	27.0 (5.1)	4.895	0.429	0.001
		dm	3.5 (1.0)	2.4 (0.7)	3.5 (0.6)	3.2 (1.3)	3.6 (1.0)	3.2 (0.4)	2.242	0.815	0.001
		vc	0.07 (0.01)	0.04 (0.01)	0.07 (0.02)	0.07 (0.03)	0.08 (0.02)	0.06 (0.01)	3.958	0.555	0.001
		sym	78.0 (10.5)	83.6 (5.1)	88.9 (5.22)	90.4 (4.3)	86.6 (9.1)	86.9 (8.6)	5.115	0.402	0.002
GM	D	tc	47.3 (7.3)	50.8 (7.3)	43.9 (5.6)	43.3 (5.3)	43.3 (4.6)	40.9 (4.8)	3.916	0.562	0.001
		dm	9.7 (1.4)	9.9 (1.9)	8.2 (2.9)	9.8 (2.9)	9.6 (1.6)	10.4 (2.8)	1.096	0.954	0.001
		vc	0.12 (0.01)	0.12 (0.02)	0.11 (0.04)	0.13 (0.03)	0.12 (0.03)	0.12 (0.03)	0.386	0.996	0.001
	N	tc	38.6 (5.8)	39.3 (4.9)	39.9 (4.0)	41.4 (2.2)	40.6 (4.2)	41.8 (1.2)	0.775	0.979	0.001
		dm	7.9 (1.9)	9.4 (1.8)	6.8 (1.8)	9.1 (2.4)	8.3 (1.8)	6.8 (2.0)	5.750	0.331	0.016
		vc	0.10 (0.02)	0.13 (0.02)	0.08 (0.03)	0.13 (0.03)	0.12 (0.02)	0.09(0.03)	8.956	0.111	0.860
		sym	85.3(10.4)	85.6(6.8)	86.3(4.1)	85.8(4.8)	89.3(3.4)	88.0(3.1)	1.124	0.952	0.001

BF, biceps femoris; GcM, gastrocnemius medialis; GM, gluteus maximus; tc, contraction time; dm, maximal radial displacement; vc, contraction velocity; sym, symmetry; Me, median, D, dominant leg; N, non-dominant leg; QD, quartile deviation; h, Kruskal–Wallis statistics; p, significance; η^2^, effect size; GK, goalkeeper; FB, fullbacks; CB, centre-backs; W, wingers; M, midfielders; F, forwards.

The contractile properties of the vastus lateralis and vastus medialis muscles are presented in Table 2. Descriptive differences were observed in contraction time (Tc) and contraction velocity (Vc) of the vastus lateralis muscle across playing positions; however, these differences should be interpreted with caution following adjustment for multiple comparisons. Before adjustment for multiple comparisons, the Kruskal–Wallis analysis identified a nominally significant positional difference with a large effect size (*η*^2^ = 0.157) for contraction velocity (Vc) of the dominant vastus lateralis muscle (*p* = 0.032). *Post hoc* analysis revealed significant differences in Vc between goalkeepers and fullbacks (*p* = 0.002), goalkeepers and forwards (*p* = 0.046), fullbacks and centre-backs (*p* = 0.008), and fullbacks and midfielders (*p* = 0.041). However, after applying the Holm–Bonferroni correction for multiple comparisons across all analyzed outcomes, this difference no longer remained statistically significant. No statistically significant positional differences were observed in any of the remaining parameters of the vastus lateralis and vastus medialis muscles (all *p* > 0.05).

**Table 2:** Contractile properties of the vastus lateralis and vastus medialis muscles according to playing position.

parameter			GK	FB	CB	W	M	F	K-W Anova			multip. comp.
			Me(QD)						h	p	η2	
VL	D	tc	23.4 (1.8)	21.5 (1.4)	23.4 (1.2)	21.0 (1.7)	21.1 (0.8)	19.6 (2.2)	9.194	0.102	0.091	
		dm	3.1 (1.1)	4.9 (0.5)	3.5 (1.1)	3.9 (0.8)	3.7 (0.3)	4.4 (0.9)	9.121	0.104	0.890	
		vc	0.07 (0.02)	0.11 (0.01)	0.07 (0.02)	0.09 (0.02)	0.09 (0.01)	0.10 (0.02)	12.219	0.032	0.157	GK vs. FB; GK vs. F; FB vs. CB; FB vs. M.
	N	tc	22.6 (2.7)	20.5 (1.4)	23.8 (0.9)	22.8 (3.4)	21.3 (1.7)	21.3 (2.1)	5.323	0.378	0.007	
		dm	3.9 (0.7)	4.5 (0.8)	4.7 (0.8)	4.2 (1.3)	4.0 (0.5)	4.9 (1.7)	1.324	0.932	0.001	
		vc	0.08 (0.02)	0.11 (0.02)	0.10 (0.02)	0.09 (0.02)	0.09 (0.01)	0.11 (0.04)	1.631	0.898	0.001	
		sym	83.5 (3.5)	92.7 (3.1)	83.3 (6.2)	82.9 (8.7)	88.2 (6.8)	88.5 (8.7)	7.426	0.191	0.053	
VM	D	tc	24.5 (0.8)	22.8 (1.8)	22.8 (1.3)	21.8 (1.3)	22.0 (1.2)	22.7 (2.8)	6.211	0.286	0.026	
		dm	7.7 (0.8)	6.4 (1.5)	6.4 (1.7)	6.5 (0.6)	5.9 (0.8)	6.5 (1.4)	3.830	0.574	0.001	
		vc	0.16 (0.02)	0.14 (0.02)	0.14 (0.03)	0.15 (0.02)	0.13 (0.02)	0.14 (0.02)	2.053	0.842	0.001	
	N	tc	23.6 (2.0)	21.6 (0.7)	23.6 (1.7)	22.7 (1.2)	22.3 (1.5)	24.7 (2.6)	3.128	0.680	0.001	
		dm	6.3 (0.4)	7.2 (0.4)	7.0 (0.6)	7.8 (1.1)	6.9 (0.9)	6.2 (1.3)	4.432	0.489	0.001	
		vc	0.14 (0.01)	0.17 (0.03)	0.15 (0.01)	0.17 (0.02)	0.16 (0.01)	0.14 (0.03)	6.469	0.263	0.032	
		sym	87.2(4.3)	85.8(3.1)	86.7(5.8)	85.9(5.4)	90.7(2.3)	84.8(2.6)	4.656	0.459	0.001	

VL, vastus lateralis; VM, vastus medialis; tc, contraction time; dm, maximal radial displacement; vc, contraction velocity; sym, symmetry; Me, median; D, dominant leg; N, non-dominant leg; QD, quartile deviation; h, Kruskal–Wallis statistics; p, significance; η^2^, effect size; GK, goalkeeper; FB, fullbacks; CB, centre-backs; W, wingers; M, midfielders; F, forwards.

## 4 Discussion

### 4.1 Main findings

Understanding the neuromuscular characteristics associated with football performance may contribute to more effective training optimization, enhanced athletic performance, and a reduction in injury risk. In this context, tensiomyography (TMG) has emerged as a promising diagnostic tool, enabling objective and non-invasive assessment of muscle contractile properties independently of an athlete's motivation or voluntary effort (22). The principal finding of the present study was that only limited positional differences in muscle contractile properties were observed among female football players. Although the lowest unadjusted *p*-value was identified for contraction velocity (Vc) of the dominant vastus lateralis muscle, this finding did not remain statistically significant after Holm–Bonferroni correction for multiple comparisons. No statistically significant positional differences were therefore observed following adjustment for multiple testing.

### 4.2 Positional differences

No statistically significant positional differences were observed in the contractile properties of the biceps femoris muscle, although descriptive differences in contraction time (Tc) and contraction velocity (Vc) were present. Similar findings have been reported in elite male football players, where only small differences in TMG-derived parameters were observed between playing positions (21).

Although no statistically significant positional differences were observed, the absence of statistical significance does not necessarily indicate that contractile characteristics are identical across playing positions, particularly given the relatively small sample size and variability within positional groups. Given the important role of the hamstring muscle group during sprinting, acceleration, deceleration, and changes of direction, as well as its high injury incidence in football, monitoring its contractile properties may still provide valuable information for performance and injury prevention purposes (28).

The lowest unadjusted *p*-value was observed for contraction velocity (Vc) of the dominant vastus lateralis muscle. Higher contraction velocities were generally observed in fullbacks, wingers, midfielders, and forwards compared with goalkeepers and centre-backs. This pattern may reflect positional differences in the demands for acceleration, sprinting, and repeated high-intensity running actions. The quadriceps muscle group plays a fundamental role in knee joint stabilization, force production during acceleration, and eccentric braking during changes of direction (29). However, this finding did not remain statistically significant after Holm–Bonferroni correction for multiple comparisons. Therefore, it should be interpreted with caution and regarded as a potential positional trend that requires confirmation in larger cohorts of elite female football players.

### 4.3 Contractile properties in the context of previous research

Maximal radial displacement (Dm) is considered an indicator of a muscle's capacity to produce a large contraction amplitude during dynamic movement (26, 30). Values between approximately 4 and 7 mm have frequently been reported as reference values in the TMG literature (31). Within this context, the female football players included in the present study demonstrated Dm values that were predominantly within the physiological range for most of the analyzed muscles. For the biceps femoris muscle, Dm values were generally within the normative range, which may indicate an adequate level of muscle elasticity and contractile function. In contrast, the gastrocnemius medialis exhibited predominantly lower Dm values, potentially reflecting greater muscle stiffness and increased muscle tone. In the vastus lateralis muscle, Dm values were primarily located at the lower limit of the physiological range or below it, which may indicate increased stiffness of the quadriceps muscle complex. Conversely, the vastus medialis displayed Dm values that largely fell within the physiological range. Higher Dm values were observed in the gluteus maximus, which may reflect lower muscle stiffness and a different functional involvement of this muscle during football-specific movement activities.

Contraction time (Tc) is commonly regarded as an indicator of muscle explosiveness, with normative values reported for football players ranging between 20 and 30 ms (22). Comparable Tc values have also been reported in football players (32). More pronounced deviations were identified in the biceps femoris and gluteus maximus muscles, where the female football players in the present study demonstrated Tc values exceeding 30 ms, suggesting slower muscle activation characteristics. Female football players in the WU17 category have previously been reported to exhibit mean Tc values of 30.2 ± 5.2 ms for the biceps femoris, whereas players in the WU19 category demonstrated values of 28.8 ± 4.6 ms (22). These findings differ from those observed in the present study. The higher Tc values recorded in our sample may be attributed to differences in training status, training methodology, neuromuscular fatigue levels at the time of testing, or variations in the phase of the competitive season. Furthermore, the influence of differences in external training load and team-specific strength and conditioning programs cannot be excluded, as these factors may substantially affect muscle contractile properties in female football players.

Differences in muscle contractile characteristics related to the specific physiological, biomechanical, and performance demands of individual sports are further supported by comparisons with athletes from other sporting disciplines. For example, contraction times exceeding 30 ms have been reported in triathletes, suggesting a greater proportion of slow-twitch muscle fibers (10), which is consistent with the predominantly endurance-based nature of the sport. Contraction time has been reported to be closely associated with the relative proportion of fast- and slow-twitch muscle fibers within a muscle (12, 13). Specifically, lower Tc values are generally associated with a greater proportion of type II muscle fibers.

### 4.4 ACL injury risk in female football players

The role of muscle mechanical and contractile properties assessed by tensiomyography (TMG) as potential risk factors for anterior cruciate ligament (ACL) injury has previously been investigated in professional male football players. The findings suggest that reduced fatigue resistance and increased stiffness of the hamstring muscles may contribute to an elevated risk of ACL injury (33). It has been well established that female football players are at a 4- to 6-fold greater risk of sustaining an ACL injury compared with their male counterparts (34, 35). Muscle imbalances between the quadriceps and hamstring muscle groups, as identified through TMG-derived parameters, may represent an additional risk factor for ACL injury in female football players (33). Furthermore, reductions in contraction velocity, fatigue resistance, and alterations in muscle tone and stiffness of the vastus medialis, vastus lateralis, biceps femoris, and gluteus maximus muscles have been associated with an increased susceptibility to ACL injury (36). Although TMG represents a promising tool for the assessment of neuromuscular muscle characteristics, its application in predicting ACL injury risk should be interpreted with caution. Muscle contractile properties constitute only one component of the multifactorial mechanisms underlying injury occurrence. Other factors, including movement biomechanics, change-of-direction technique, fatigue status, and individual anatomical predispositions, also play substantial roles in ACL injury risk. Moreover, the current body of evidence regarding the application of TMG in women's football remains limited and highlights the need for further longitudinal investigations.

### 4.5 Potential explanations for the lack of positional differences

Several authors have suggested that modern football places substantial demands on neuromuscular performance across all playing positions, which may contribute to relatively similar muscle contractile characteristics across playing positions. The absence of statistically significant positional differences observed in the present study may be consistent with the similar movement demands characteristic of contemporary football. However, this interpretation should be considered with caution because the findings may equally reflect the limited statistical power of the study, particularly the small goalkeeper subgroup, and the substantial inter-individual variability in TMG-derived parameters (5, 37). Modern football is characterized by repeated accelerations, decelerations, and high-speed running actions that are executed across all playing positions (38). However, given the relatively small sample size and the variability observed within positional groups, this interpretation should be considered with caution. The relatively small sample sizes within several positional groups, particularly the goalkeeper group, may have reduced the statistical power of the analyses and increased the likelihood of Type II error. Therefore, the absence of statistically significant differences should not be interpreted as evidence of the absence of true positional differences, but rather as findings that warrant confirmation in larger cohorts of elite female football players.

Although previous investigations have reported greater locomotor and mechanical demands in wide playing positions, recent evidence suggests that positional differences may be becoming less pronounced due to tactical evolution and the increasing physical demands imposed across all playing roles (39). Furthermore, tactical organization and match context may currently exert a greater influence on physical and neuromuscular demands than the nominal playing position itself (40). Another possible explanation for the absence of clear positional differences may be that TMG-derived parameters are less sensitive to subtle neuromuscular variations among elite players exposed to similar training loads. In addition, individual variability in movement strategies and neuromuscular characteristics may have contributed to the lack of distinct positional profiles (41). Given the limited evidence currently available in women's football, further research is required to determine whether position-specific differences in muscle contractile properties are inherently less pronounced in female football players compared with their male counterparts. Despite the absence of statistically significant positional differences after adjustment for multiple comparisons, TMG may still provide valuable information regarding individual neuromuscular status, fatigue monitoring, and injury prevention strategies in female football players.

### 4.6 Limitations

The present study has several limitations that should be considered when interpreting the findings. First, the relatively small sample size may limit the generalizability of the results to a broader population of female football players across different competitive levels. However, the study included all eligible elite female football players available during the data collection period; therefore, a formal *a priori* power analysis was not performed. Another limitation is the low number of goalkeepers (*n* = 4), which may have affected the statistical power of the analyses and reduced the sensitivity to detect potential differences between this playing position and the other groups

Another limitation is the cross-sectional design of the study, which does not allow for the assessment of long-term neuromuscular adaptations throughout a competitive season or training process. In addition, several potentially important confounding factors influencing muscle contractile properties were not controlled. Although age, body height, body mass, and football experience were recorded, external training load, GPS-derived locomotor demands, strength-training history, and cumulative match exposure were not systematically assessed. Furthermore, menstrual cycle phase and hormonal contraceptive use were not recorded or standardized, although hormonal fluctuations may influence neuromuscular function and muscle contractile properties in female athletes. Consequently, the potential influence of these variables on TMG-derived parameters cannot be excluded. Only selected lower-limb muscle groups were evaluated, while other muscles substantially involved in football-specific movements were not assessed. Finally, the relationships between TMG-derived parameters and performance-related indicators, such as sprint speed, change-of-direction ability, and lower-limb explosive power, were not examined. Future longitudinal studies should address these limitations to provide a more comprehensive understanding of neuromuscular adaptations in elite female football players.

### 4.7 Future directions

Future research should include larger samples of female football players from different competitive levels and focus on the longitudinal monitoring of muscle contractile properties throughout the competitive season. Furthermore, investigating the relationships between TMG-derived parameters, external match load, neuromuscular fatigue, and performance-related indicators would provide a more comprehensive understanding of the factors influencing neuromuscular function in female football players. Another promising direction is the examination of the effects of the menstrual cycle on neuromuscular characteristics, as well as the application of tensiomyography for the prediction and prevention of muscle and ligament injury risk.

## 5 Conclusion

The findings indicate only limited positional differences in the contractile properties of lower-limb muscles among female football players. Although the lowest unadjusted *p*-value was observed for the contraction velocity of the dominant vastus lateralis muscle, this finding did not remain statistically significant after Holm–Bonferroni correction for multiple comparisons. The absence of statistically significant positional differences may reflect the relatively similar neuromuscular characteristics of elite female football players; however, this interpretation should be considered with caution given the limited statistical power of the study, particularly the small goalkeeper subgroup, and the substantial inter-individual variability observed within positional groups.

Despite the absence of statistically significant positional differences after adjustment for multiple comparisons, tensiomyography appears to be a promising non-invasive tool for individualized neuromuscular monitoring in female football players and may support training decision-making when interpreted alongside performance, workload, and clinical assessments.

## Data Availability

The datasets presented in this study can be found in online repositories. The names of the repository/repositories and accession number(s) can be found below: https://zenodo.org/records/20681856.
